# Muscle paresis and passive stiffness: Key determinants in limiting function in Hereditary and Sporadic Spastic Paraparesis

**DOI:** 10.1016/j.gaitpost.2011.09.018

**Published:** 2012-02

**Authors:** Jon Marsden, Gita Ramdharry, Valerie Stevenson, Alan Thompson

**Affiliations:** a School of Health Professions, Peninsula Allied Health Centre, University of Plymouth, Derriford Road, PL6 8BH, United Kingdom; b School of Rehabilitation Sciences, St. George's University of London and Kingston University, Cranmer Terrace, SW17 ORE, United Kingdom; c Department of Brain Repair and Rehabilitation Institute of Neurology, Queen Square, London WC1N 3BG, United Kingdom

**Keywords:** Hereditary Spastic Paraparesis, Stiff legged gait, Spasticity, Stiffness, Walking, Paresis

## Abstract

**Background:**

People with Hereditary and Sporadic Spastic Parapresis (SP) walk with a stiff legged gait characterised by a lack of knee flexion.

**Objective:**

We investigated the relationship between lower limb strength and stiffness and knee flexion during swing phase while walking in 20 people with SP and 18 matched controls.

**Methods:**

Maximal isometric strength was measured using a dynamometer. Passive stiffness and spasticity was assessed during motor-driven slow (5°/s) and fast (60°/s) stretches at the ankle and knee while the subject was relaxed or preactivating the muscle. Walking was assessed using 3D motion analysis.

**Results:**

Isometric muscle strength was decreased in people with SP with over a 50% reduction in strength being found in the ankle dorsiflexors. Passive stiffness, assessed during slow stretches, was 35% higher in the plantarflexors in people with SP (*P* < 0.05). Faster stretches induced large stretch evoked muscle activity and over a 110% increase in stiffness at the ankle and knee in people with SP reflecting the presence of spasticity (*P* < 0.05). However, stretch reflex size and stiffness was similar between the groups following identical stretches of the pre-activated muscle (*P* > 0.05). Lower knee flexion during swing phase was associated with reduced knee flexion velocity at the end of stance phase which in turn was associated with reduced plantarflexor strength and increased passive stiffness in the knee extensors.

**Conclusions:**

The relative importance of muscle paresis and passive stiffness in limiting walking in SP suggests that these impairments should be the target of future therapies.

## 1 Introduction

Hereditary Spastic Paraparesis (SP) is an heterogeneous degenerative condition. In the type I or uncomplicated presentation people present with predominately lower limb paresis and spasticity with ∼40% of people showing an additional reduction in vibration sense. In the type II or complicated forms there may be added signs, for example, myopathy, cerebellar ataxia or dementia [1,2].

Pathological studies in spastic paraparesis reveal a dying back axonal degeneration of the corticospinal tracts, fasciculus cuneatus and spinoceberebellar tracts with additional degeneration of the corpus callosum in people with dementia [3]. Both autosomal, recessive and X linked forms of inheritance have been described with abnormalities in axonal transport being implicated in the pathogenesis of the most common form caused by mutation of the spastin gene [4]. Spastin mutations are also seen in ∼13% of people with sporadic onset of spastic paraparesis restricted to the legs [5,6].

Difficulties with walking and standing balance are commonly reported in people with spastic paraparesis. People with Hereditary and Sporadic SP trend to walk with a stiff-legged gait characterised by a reduction in knee flexion during swing phase, often with the addition of increased hip adduction during the swing phase [7,8]. Reduced knee flexion can lead to an increased incidence of trips and falls and to compensatory strategies such as leg circumduction that can greatly increase the effort of walking.

Typically such patterns of walking are felt to be mainly caused by the presence of spasticity [9,10]. Indeed excessive muscle activity of the knee extensors, such as rectus femoris, during preswing and swing phase could limit knee flexion during swing phase. However, studies modelling the contributions of individual muscles during walking suggest that there may be multiple factors contributing to a particular gait pattern. The amplitude of knee flexion in swing phase, for example, is strongly dependent on the velocity of knee flexion at the end of stance phase [11]. The degree of knee velocity in turn depends on the activity of the ankle plantarflexors and hip flexors which are responsible for initiating swing phase [12]. Therefore, factors that affect swing phase initiation such as muscle paresis of the hip flexors or plantarflexors can cause a stiff legged gait.

Understanding the underlying cause of a given pattern of walking will allow clinicians to more effectively target therapies. This study examined the relationship between the lack of knee flexion in people with spastic paraparesis and their underlying impairment.

## 2 Methods

### 2.1 Recruitment criteria

Twenty people with Spastic Paraparesis (SP) were recruited from the Hereditary Spastic Paraplegia support group, UK and the Neurogenetics and Spasticity clinics at the National Hospital for Neurology and Neurosurgery, London, UK. People were included if they had a clinical diagnosis of type 1 spastic paraparesis and were able to walk at least 100 m with or without a walking aid. Exclusion criteria included the presence of additional neurological or orthopaedic impairments. None of the participants were on regular anti-spasticity medication or had received botulinum toxin injections within the last 3 months. People matched for age, gender and height with no history of neurological or orthopaedic impairment were recruited from colleagues and spouses/friends of people with SP to act as a control group. People participated with informed written consent and the approval of the local ethics committee in accordance with the Declaration of Helsinki.

### 2.2 Clinical outcome measures

Clinical measures of three functional movements were taken. Maximal and normal walking speed and cadence was measured over 10 m; gait aids were used as required and a 2 min rest was given after each trial. Timed sit to stand (×5) was assessed as participants stood up and down from a chair (50 cm height) with their arms folded. Balance was assessed using the Berg balance scale.

### 2.3 Measurement of walking

Three-dimensional joint kinematics and kinetics were measured via markers placed on standardised bony landmarks and wands (Codamotion, Charnwood dynamics, UK) while the person walked on a customised walkway containing two embedded force plates (9286AA Kistler, Instruments Ltd., Hampshire, UK). People used a gait aid as required (1 stick *n* = 6; 2 sticks/crutches *n* = 2) but without the use of any external electrical stimulation or use of any orthotic. A total of 3 steps with either leg landing on a single force plate were recorded. Control participants walked at a matched speed and cadence. Lines at the start of the walkway indicated the desired step length and auditory cues about the required step frequency were provided via a metronome, practice trials were provided prior to recording the data. Data was AD converted (1 kHz for EMG and 200 Hz for force plate and motion analysis data) for off line analysis.

### 2.4 Measures of impairment

Measures of lower limb impairment were taken after the assessment of walking to avoid muscle fatigue associated with the tests impacting on the pattern of walking. A 20 min rest was provided between the walking test and impairment measures. In all cases the right leg was measured.

*Isometric strength* was measured using a dynamometer (Biodex Systems 3, IPRS Mediquipe, UK). Agonist–antagonist pairs at the hip, knee and ankle were measured in standardised positions (see supplementary material). The axis of the motor was aligned with the axis of the joint and the proximal segment fixed. The maximal voluntary contraction (MVC) was recorded twice and the applied torque was recorded (2 kHz AD sampling rate).

*Limb stiffness* was measured by applying ramp and hold stretches to the ankle plantarflexors and the knee extensors (Biodex Systems 3, IPRS Mediquipe, UK). Stretches had a 5° amplitude with a peak velocity of either 5 or 60°/s with a return velocity of 5°/s. Six stretches per velocity condition were recorded with a 6.5 s inter-stretch interval. The order of the conditions was randomised between participants. Stretches were either delivered with the participant resting or pre-activating the muscle of interest to achieve a torque of 10 Nm. This torque level corresponded to approximately 10% of the maximal voluntary contraction (MVC) achieved by the people with SP. Additionally, control subjects pre-activated their muscle to the same percentage of their MVC that was achieved in their matched participant with SP. Surface electromyography (EMG, MT8 Telemetry, MIE, Leeds, UK) was recorded from the medial head of gastrocnemius, tibialis anterior and rectus femoris and medial hamstrings at mid thigh level with an inter-electrode distance was 2.5 cm. During ankle stretches the participant was supine with the knee extended and the ankle in plantigrade. During knee extensor/flexor stretches the participant was supine with the hip extended and the knee flexed by 90°. The contralateral leg was supported in extension. The torque, position, velocity and surface EMG were AD converted at 2 kHz (Power 1401, Spike 2, Version 5, CED Electronics, Cambridge, UK) and stored for off-line analysis.

## 3 Analysis

### 3.1 Walking

Three dimensional joint angles, internal joint moments and power normalised to body weight were calculated using inverse dynamics (CODAmotion, Leister, UK). One step cycle including ipsilateral and contralateral foot on and foot off was defined from the vertical ground reaction force and the horizontal and vertical acceleration of the toe and heel markers. Each gait cycle was normalised to 100% and 3 cycles for each leg were averaged. Preswing was defined as the period of double stance between contralateral foot down and ipsilateral foot off.

The peak knee flexion and extension amplitude in swing phase and the peak knee flexion velocity in preswing were determined. Peak ankle and hip power generation and knee extensor torque during pre-swing was assessed.

*Isometric strength*: The MVC was defined as the peak difference between maximal and baseline torque and was normalised to the body weight.

*Limb stiffness*: Imposed stretches were aligned to the onset of the stretch; the first stretch was omitted to allow for the effects of thixotropy and the final 5 stretches were averaged. The average torque and position was calculated over a 100 ms period prior to the onset of the stretch and immediately following the cessation of the stretch. Stiffness was defined as:

$$\text{Stiffness}=\frac{\Delta \;\text{Torque}}{\Delta \;\text{Position}}$$

Stiffness was normalised to the body weight. Surface EMG was filtered (30 Hz low pass filter) and rectified. A stretch-evoked response occurred if the EMG signal moved above a level of the baseline mean + 4 standard deviations within a 25–125 ms post stretch window. The mean amplitude between the onset and offset of activity, when the EMG fell rose and below this level, was calculated.

### 3.2 Statistical analysis

Differences in strength and stiffness between the SP and control group were compared using an unpaired two-tailed *t*-test. Although walking speed during the walking test was not significantly different between groups the control group did tend to walk faster despite auditory and visual cues about step length and cadence. Therefore measures of walking were analysed using an analysis of covariance with walking speed as a covariate. A Bonferroni correction was applied to account for multiple comparisons during the assessment of walking (*n* = 6), muscle strength (*n* = 8) and stiffness (*n* = 4 per muscle). The relationship between impairment and gait-related variables and peak to peak swing phase knee amplitude and peak knee flexion velocity in preswing phase were assessed using a Pearson correlation. Data was felt to be significant if *P* < 0.05.

## 4 Results

Twenty people with spastic paraparesis were compared to 18 healthy participants matched for gender, age, height and weight (Table 1). A family history was present in 15 of the people with SP with five of these having a genetic diagnosis (SPG4 *n* = 4 and X-linked *n* = 1). The reported age of symptom onset was 27.4 yrs (18.2 ± standard deviation).

People with SP had a significantly reduced normal and maximal walking speed and cadence. They were slower standing up/sitting down and had lower scores on the Berg balance scale indicating impaired standing balance (Table 1).

### 4.1 Measures of impairment

#### 4.1.1 Isometric muscle strength

**Fig. 1:**
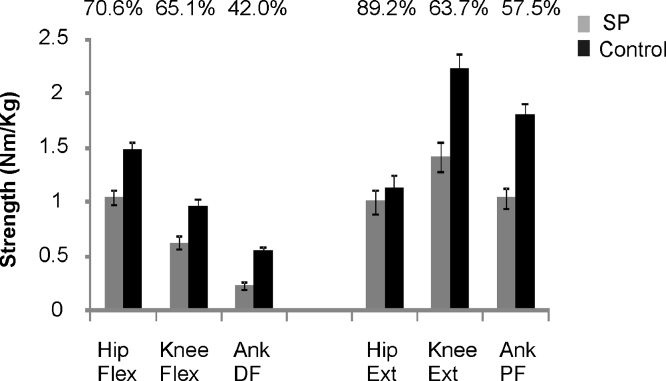
Isometric strength. Mean ± standard error of the mean (SEM) indicated. Above each column is the strength of the SP group expressed as a percentage of the controls highlighting the relative reduction in strength in the more distal muscle groups.

Isometric muscle strength was significantly reduced in people with SP in every muscle group assessed (*P* < 0.01). The reduction in muscle strength, relative to controls, was greater in distal muscle groups (Fig. 1).

#### 4.1.2 Muscle stiffness and spasticity

**Fig. 2:**
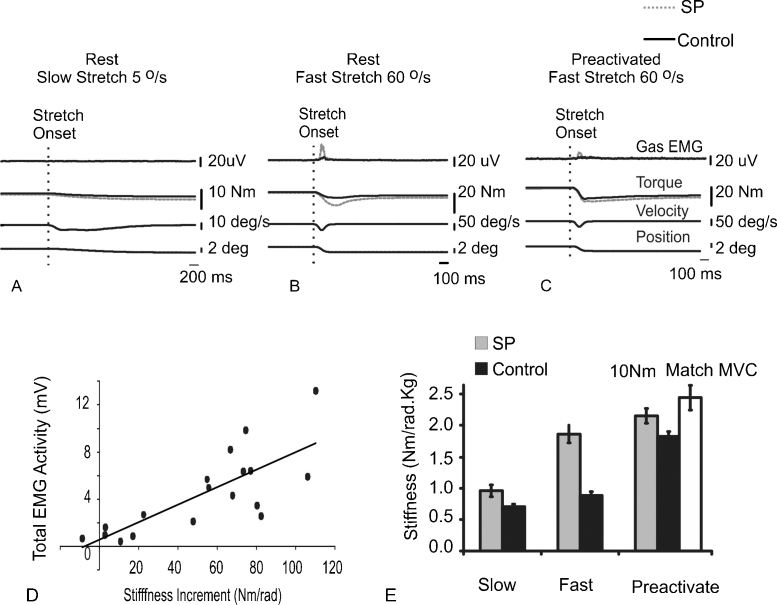
Change in ankle stiffness with different speeds of stretch. The grand average response to ramp stretches of the ankle into dorsiflexion is indicated in A–C. Stretches were applied at different speeds (5°/s A or 60°/s B and C) with the participant either resting (A and B) or preactivating the ankle plantarflexors (C). (D) The relationship in the SP group between the stretch evoked gastrocnemius EMG activity following a fast stretch and the increase in stiffness between the fast and slow stretch at rest. (E) Differences in stiffness following different stretches at the ankle. The grey bar indicates the stiffness recorded when the controls preactivated with plantarflexors to the same percentage of their maximal voluntary contraction as seen in the matched SP participants (mean ± SEM is indicated).

Slow stretches (5°/s) were not associated with any change in muscle activity; thus the stiffness is thought to reflect a passive, non-reflexive component (Fig. 2A). In the plantaflexors the passive stiffness was significantly higher in people with SP (*t* = 2.6, *P* = 0.01; Table 2). There was no difference in knee extensor stiffness associated with a slow stretch (*t* = 0.02, *P* > 0.05; Table 2).

Faster stretches (60°/s) resulted in short latency stretch-evoked muscle activity in people with SP that resulted in a further increase in joint stiffness (Fig. 2B, *n* = 20/20 ankle plantarflexors, *n* = 17/20 knee extensors). Stretch-evoked activity was less frequently seen in the control group (*n* = 16/18 ankle plantarflexors *n* = 8/18 knee extensors). The relative increase in stiffness compared to that seen with the slow stretch (fast–slow stretch) correlated with the stretch reflex amplitude in the SP group (*R*^2^ = 0.61; Fig. 2D). The relative increase in stiffness with the fast stretch was significantly higher in people with SP for both muscle groups (ankle *t* = 4.4, *P* < 0.001; knee *t* = 5.5, *P* < 0.001; Table 2).

When participants pre-activated the muscle prior to the fast (60°/s) stretch there was no difference in the total stiffness between the groups (Fig. 2C and Table 2). This was seen regardless of whether the control group pre-activated the muscle to the same torque level as the SP group (10 Nm) or the same percentage of the MVC as the matched SP participant.

### 4.2 Factors affecting stiff-legged gait

**Fig. 3:**
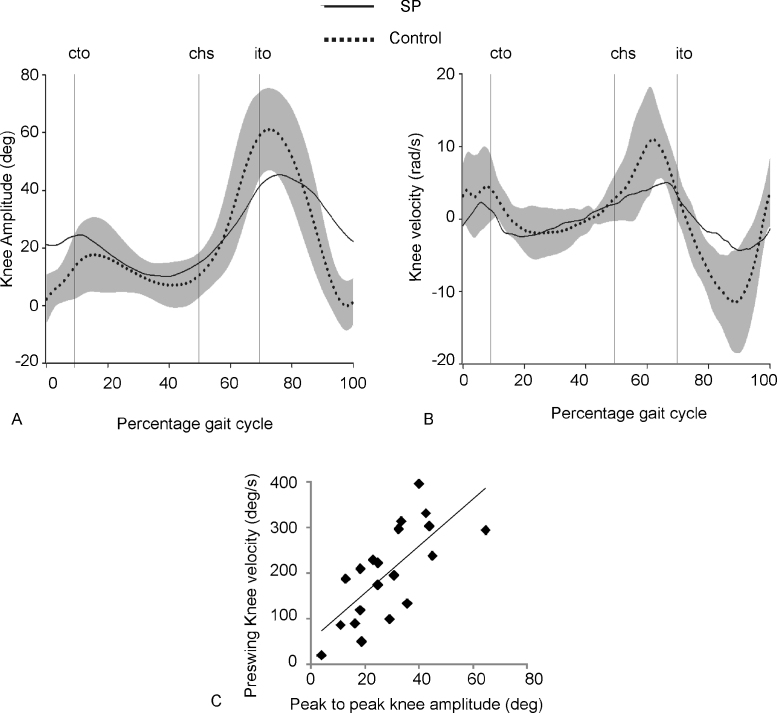
Stiff legged gait in SP. Grand average knee amplitude (A) and knee velocity (B). Grey bars indicate 2 standard deviations of the control group. Cto, contralateral toe off; chs, contralateral heel strike; ito, ipsilateral toe off. (C) Association between peak knee velocity during preswing and peak to peak knee motion during swing phase.

People with SP had reduced knee flexion and knee extension in swing phase (Fig. 3A) resulting in a significant decrease in peak to peak knee amplitude (*F*(2,35) = 62, *P* < 0.001; Table 1). People with reduced knee flexion velocity in pre-swing tended to show a reduction in knee motion during swing phase (*R*^2^ = 0.51; Fig. 3B and C).

During pre-swing, peak ankle power generation tended to be reduced (*F*(2,35) = 4.8, *P* < 0.05) and knee extensor torque was significantly increased (*F*(2,35) = 64.1, *P* < 0.001) in people with SP. Peak hip flexor power generation was significantly increased in the SP group when co-variation due to walking speed was accounted for (*F*(2,35) = 9.3, *P* < 0.005; Table 1). The reduction in ankle power and the increase in knee extensor torque was associated with a reduction in knee flexor velocity in preswing (ankle power *R*^2^ = 0.24, *F*(1,19) = 5.5, *P* < 0.05; knee extensor moment *R*^2^ = 0.54, *F*(1,19) = 20.8, *P* < 0.001).

The ankle power generation was correlated to the isometric ankle plantarflexion strength (*R*^2^ = 0.37, *F*(1,19) = 10.4, *P* < 0.005) while the size of the knee extensor moment was correlated with the degree of passive stiffness in the knee extensors (*R*^2^ = 0.42, *F*(1,19) = 12.9, *P* < 0.005). In contrast there was no relationship between the knee extensor moment and either the increase in knee extensor stiffness measured following fast (60°/s) stretches at rest (*R*^2^ = 0.17, *F*(1,19) = 3.6, *P* > 0.05) or the total stiffness recorded after a fast (60°/s) ramp stretch of the pre-activated knee extensors (*R*^2^ = 0.05, *F*(1,19) = 0.9, *P* > 0.36). In a multiple regression analysis the isometric ankle plantarflexion strength and passive stiffness of the knee extensors accounted for 52% of the variance in peak knee flexion velocity during swing phase (*F*(2,19) = 9.3, *P* < 0.005) and 50% of the variance in peak to peak knee amplitude during swing phase (*F*(2,19) = 8.6, *P* < 0.005).

## 5 Discussion

Knee flexion amplitude in swing phase was related to the size of the knee velocity at the end of stance phase and in turn to paresis of the ankle plantarflexors and passive stiffness in the knee extensors. In contrast there was no association with measures of stretch reflex hyperexcitability or spasticity [13].

Muscle paresis affected multiple muscle groups in people with SP, being greatest in the ankle dorsiflexors. Muscle paresis may reflect both a decrease in corticospinal drive and secondary muscle atrophy [14] and is often the main determinant in limiting functional movements in people with an upper motor neuron lesion [15,16]. Indeed there was a significant negative correlation between the degree of strength in the knee extensors and the sit-to-stand time (*R*^2^ = 0.47) and a positive correlation between the strength in the ankle plantarflexors and normal walking speed (*R*^2^ = 0.61). The presence of significant muscle weakness that can limit functional movements in SP is in contrast with the view that paresis is relatively mild in this patient group and that function is mainly limited by spasticity [10]. This may in part reflect the fact that active movements as opposed to objective tests of muscle strength have been assessed to date.

We found that a high knee extensor moment during pre-swing limited swing phase knee flexion and was associated with a higher degree of passive stiffness in the knee extensors. Increases in passive stiffness could reflect changes in the connective tissue, muscle architecture and/or intrinsic muscle proteins [17] and have been reported in other conditions affecting the central nervous system such as multiple sclerosis or stroke [18–21]. The change in passive stiffness was more marked for the plantarflexors than for the knee extensors; this may reflect different patterns of use and differences in the amount or pattern of intramuscular connective tissue. However, in those people with SP an above average passive stiffness in the knee extensors was associated with a further limitation in knee flexion.

Hyperexcitable reflexes were seen when higher velocity stretches were applied at rest in people with SP, this is a hallmark of spasticity [13]. Stretch reflex size can decrease following a stretch 2–10 s earlier, termed post activation depression. Post activation depression can be reduced in people with spasticity [22] and given the inter-stretch interval of 6.5 s, this could also contribute to the high stretch reflex size observed. The increase in stretch-evoked muscle activity and reduction in post-activation depression reflects in part a decrease in inhibitory activity within spinal cord circuits [23]. The degree of reciprocal inhibition, for example, from the ankle dorsiflexors to the ankle plantarflexors is reduced in people with spasticity [24]. In healthy participants the level of reciprocal inhibition is not static. With the onset of contraction of the plantarflexors the reciprocal inhibition of that muscle decreases [25]. This could explain why, compared to the resting condition, identical stretches of a pre-activated muscle resulted in EMG evoked activity and total stiffness that was similar between groups. Such a normalisation of muscle stiffness and stretch reflex activity in people with spasticity [26,27] raises the question as to the role of spasticity in limiting movement, particularly if the stretched muscle is pre-activated such as during an eccentric contraction.

No relationship between spasticity of the knee extensors and the degree of knee flexion was seen. Stretch reflex size is normally modulated by postural set and the phase of walking [28]. In people with spasticity such modulation is decreased [28]. Therefore, a lack of correlation between stretch reflex size recorded in supine and parameters of walking may reflect differences in stretch reflex properties during functional tasks such as walking. There may also be differences between how the response to unexpected perturbations (as delivered in the current study) and expected perturbations as occurs during voluntary movement. Stretch reflexes were only recorded at one velocity (60°/s). Non-linearity in response to different stretch velocities may further mean that the contribution of stretch reflexes at the velocities achieved while walking (∼140°/s) was underestimated.

The current work found that the limitation in knee flexion velocity and peak to peak knee amplitude during swing phase was associated with muscle paresis and passive stiffness; explaining ∼50% of the variance, rather than static measures of spasticity. This is in keeping with recent work showing that excessive activation of the rectus femoris in pre-swing while walking is less common in children with SP compared to children with cerebral palsy and spastic diplegia [8,29]. This study has only investigated associations between variables and it remains unclear whether ankle strength and knee extensor passive stiffness actually cause a reduction in knee movement while walking. However, this study highlights the need to determine muscle strength objectively, to assess the contribution of stretch reflexes to limb stiffness in both the resting and active participant and to differentiate between limb stiffness caused passive stiffness and spasticity; this will have a direct impact on potential therapeutic approaches. While spasticity may be amenable to pharmacological interventions; physical interventions such as stretching or splinting may be more applicable to target changes in passive stiffness.

## Figures and Tables

**Table 1:** Demographic data and clinical characteristics of the SP and control groups.

	SP	Control
Age (yrs)	49 ± 13.9	48.1 ± 13.4
Height (cm)	172.7 ± 9.5	174.6 ± 5.5
Weight (kg)	75.5 ± 13.4	73.4 ± 9.8
Gender (males)	12	11
10 m walk normal speed (speed m/s and cadence steps/min)	Speed 0.89 ± 0.29 Cadence 104.0 ± 19.7	Speed 1.46 ± 0.18^a^ Cadence 117.3 ± 7.4^a^
10 m walk maximal speed (speed m/s and cadence steps/min)	Speed 1.22 ± 0.48 Cadence 122.5 ± 27.4	Speed 2.13 ± 0.31^a^ Cadence 146.5 ± 15.7^a^
Sit to stand time ×5 (s)	22.5 ± 16.8	8.7 ± 1.8^a^
Berg balance scale	49.5 ± 13.5	56.0 ± 0^a^
Walking speed during the clinical test (m/s)	0.86 ± 0.4	1.10 ± 0.4
Peak to peak knee motion in swing (°)	28.6 ± 3.2	61.7 ± 2.2^a^
Peak knee flexion velocity in preswing (rad/s)	3.5 ± 0.4	5.7 ± 0.4^a^
Peak ankle power preswing (W/kg)	22.7 ± 0.3	1.5 ± 0.2^a^
Peak knee extensor moment preswing (Nm/kg)	0.43 ± 0.06	0.18 ± 0.03^a^
Peak contralateral (left) hip flexor power loading phase (W/kg)	0.22 ± 0.02	0.19 ± 0.03

Mean ± standard deviation is indicated except for the Berg balance scale where the median ± interquartile range is shown.

a Significant difference between groups.

**Table 2:** Measures of stiffness (Nm/rad kg) in response to ramp stretches that stretch the ankle plantarflexors or knee extensors.

Condition	Ankle Plantarflexors		Knee extensors	
	HSP	Control	HSP	Control
Slow stretch (5°/s) at rest	0.95 ± 0.08^a^	0.71 ± 0.03	0.33 ± 0.03	0.33 ± 0.04
Fast stretch (60°/s) at rest	1.90 ± 0.16^a^	0.90 ± 0.06	0.87 ± 0.09^a^	0.39 ± 0.03
Fast–slow stretch at rest	0.95 ± 0.15^a^	0.19 ± 0.06	0.54 ± 0.08^a^	0.06 ± 0.02
Fast stretch (60°/s) preactivated to 10 Nm	2.2.1 ± 0.12	1.87 ± 0.12	1.21 ± 0.11	1.29 ± 0.09
Fast stretch (60°/s) preactivated to the same percentage MVC	NA	2.49 ± 0.17	NA	1.63 ± 0.18

Mean ± SEM is indicated.

a Significant difference between the HSP and control group following a Bonferroni correction.
