# Supplementary material for: Muscle paresis and passive stiffness: Key determinants in limiting function in Hereditary and Sporadic Spastic Paraparesis
Source: Gait Posture. 2012 Feb;35(2):266–71. doi: 10.1016/j.gaitpost.2011.09.018 (PMC3657152; doi:10.1016/j.gaitpost.2011.09.018)
Supplement: Supplementary file 1 [file mmc1.zip › mmc1.docx]

Supplementary Data

| **Muscle Group** | **Position** |
| --- | --- |
| *Measures of Isometric Strength* | |
| Ankle Plantarflexors / dorsiflexors | Ankle in plantigrade with knee extended  Fixation given at the lower and upper leg, pelvis and shoulder girdle |
| Knee Extensors/ Flexors | Sitting with hips and knee at 90  Fixation at thigh and trunk |
| Hip Flexors/ Extensors | Supine with hip in 30 flexion  Fixation at the trunk and pelvis |
| Hip Abduction /Adduction | Supine with hip in neutral and knee extended  Fixation at the pelvis |
| *Measures of muscle stiffness* | |
| Ankle Plantarflexors / dorsiflexors | Ankle in plantigrade with knee extended  Fixation given at the lower and upper leg, pelvis and shoulder girdle |
| Knee Extensors | Supine with 10 deg hip flexion and the knee at 90  Fixation at thigh and trunk |
| Knee Flexors | Sitting with hips and knee at 90  Fixation at thigh and trunk |

Supplementary Material: The positions and fixation points used for the measurement of isometric strength and muscle stiffness. In all cases the distal limb segment was attached to the maniplandum and the motor axis aligned with the axis of the joint.
